# Neural Oscillatory Markers of Voluntary Task Switching: Proactive Engagement of Self‐Directed Control in Children and Adults

**DOI:** 10.1111/desc.70073

**Published:** 2025-09-08

**Authors:** Nicolas Chevalier, Aurélien Frick

**Affiliations:** ^1^ Department of Psychology University of Edinburgh Edinburgh UK; ^2^ Department of Psychology and Neuroscience University of St Andrews St Andrews UK

**Keywords:** cognitive control, EEG, executive function, proactive control, self‐directed control

## Abstract

Cognitive control shows two main developmental trends: greater self‐directedness (i.e., children need less external scaffolding) and greater proactiveness (i.e., children increasingly anticipate and prepare for upcoming cognitive demands). The present study examined potential links between these major developmental transitions. Specifically, it used EEG oscillations to investigate the extent to which children, like adults, engage self‐directed control in a proactive fashion, and whether age‐related changes reflect progress in task selection, motor preparation, or both. Five–6‐year‐olds, 9–10‐year‐olds, and adults performed a voluntary task‐switching paradigm in which visual support for past actions was manipulated. Both children and adults showed greater frontolateral delta/theta power and lower central mu power on switch than repeat trials, but visual support differentially affected these oscillatory markers across age groups. Children already engage self‐directed control proactively from 5 to 6 years of age in the voluntary task‐switching paradigm, albeit differently than adults, suggesting close links between self‐directed and proactive control developments.

**Summary:**

- Neural oscillatory markers of proactive control are examined in 5–6‐year‐olds, 9–10‐year‐olds, and adults in the voluntary task‐switching paradigm.
- Both children and adults engaged in proactive task selection and motor preparation, as evidenced by frontolateral delta/theta power and mu suppression, respectively.
- Children already engage self‐directed control proactively from 5–6 years of age in the voluntary task‐switching paradigm, albeit differently than adults.

## 1 Introduction

Increasingly flexible, complex and independent behaviour throughout childhood is supported by the protracted development of cognitive control—the goal‐directed regulation of attention, thoughts, and action. Research on cognitive control development has largely focused on domains of control (i.e., working memory, inhibitory control, and shifting) but also shown general, cross‐domain trends (or transitions) in the way control is engaged. Specifically, as children grow older, they engage cognitive control more *self‐directedly*, relying progressively less on external aid and agents (e.g., caregivers), and more *proactively*, better anticipating and preparing for upcoming events (e.g., Chatham et al. 2009; Frick et al. 2019; Lucenet and Blaye 2014; Snyder and Munakata 2010). Yet, these two major changes have been studied in isolation, and little is known about their potential relation. The present study investigated whether self‐directed cognitive control development relies on greater proactiveness with age.

In most Western countries, young children often engage cognitive control in an externally driven (or exogenous) fashion, with external aid or agents scaffolding goal‐directed behaviour (Alcalá 2023). That is, they need external support to identify, maintain, and use goals to guide their behaviour, such as explicit instructions from their parents to start cleaning up their room. With age, children can do so by relying on increasingly subtle contextual cues (e.g., a simple parental glare rather than explicit instructions) and eventually without being externally prompted (Barker and Munakata 2015; Chevalier and Blaye 2009; Frick and Chevalier 2023; Munakata et al. 2012), hence showing greater self‐directed (or endogenous) engagement of cognitive control (self‐directed control, for short). This developmental pattern echoes similar age‐related transitions from co‐regulation to self‐regulation (e.g., Wesarg‐Menzel et al. 2023) and from extrinsic to intrinsic motivation (Deci and Ryan 2000). It is also consistent with Vygotsky's 1978 *internalisation* of high‐order cognitive functions, through which children progressively internalise cultural values and regulatory practices that were initially supported by external agents (e.g., caregivers, teachers).

Self‐directed control differs from externally driven control in that relevant goals cannot be exclusively identified and maintained in working memory by monitoring and processing external cues. Instead, abstract representations (e.g., about what cleaning up a room entails) seem critical to sequence goals and actions (e.g., putting toys in chest, books on shelf, etc.; Munakata et al. 2012; Orr and Banich 2014). Abstract representations, however, may not be sufficient to decide on what to do next. One may also need to keep track of past and future actions and events to determine, based on this contextual information, which goals and actions are most relevant (e.g., if a child has been playing for a while and it will soon be dinner time, they may decide to start cleaning up; Frick et al. 2022; Frick and Chevalier 2023). Consistently, facilitating context‐tracking through provision of visual support for previous actions improves self‐directed control engagement in children (Frick et al. 2022).

If self‐directed control entails keeping track of past actions/events and *anticipating future ones*, self‐directed control may be intrinsically related to proactive engagement of cognitive control (proactive control, for short; for a similar point, see Mahy et al. 2015). Proactive control consists of anticipating and preparing for upcoming tasks or events by getting ready to process task‐relevant information and select task‐relevant responses (e.g., Braver 2012). As it minimises subsequent conflict with task‐irrelevant information, it generally yields greater behavioural performance (e.g., faster responses) than reactive, in‐the‐moment resolution of conflict that has already arisen, when upcoming tasks can be reliably predicted (e.g., Chevalier et al. 2015; Marklund and Persson 2012). Preschoolers, however, tend to mostly engage control reactively, although they can already engage it proactively when encouraged (e.g., Chevalier et al. 2015; Gonthier and Blaye 2022; Yanaoka et al. 2022). With age, children engage proactive control more flexibly, systematically, and consistently (e.g., Chatham et al. 2009; Elke and Wiebe 2017; Hayre et al. 2022; Kubota et al. 2020), although they tend not to show as much behavioural benefit (i.e., response time reduction) as adults do (e.g., Chevalier et al. 2020; Niebaum et al. 2020).

An open question is whether self‐directed control development may be driven at least in part by greater proactiveness with age. On the one hand, the very nature of self‐directed control (i.e., keeping track of past actions and anticipating new ones) may encourage even younger children, who generally rely on reactive control, to engage in self‐directed control proactively. On the other hand, the extra demands related to self‐directedness (relative to externally driven control; Frick et al. 2021) may lead children, especially younger ones, to fall back on the less mature, reactive form of control (to reduce cognitive demands). This question can be explored experimentally using the voluntary task‐switching (VTS) paradigm, one of the most widespread measures of self‐directed control in adults (Arrington et al. 2014; Arrington and Logan 2004). VTS requires freely selecting which of two tasks (e.g., colour‐ or shape‐matching) to perform on each trial, without any external information (i.e., no task cue signals which task to perform). Thus, task selection refers to the decision to perform one task over the other, which involves activating or upweighting the activation of the corresponding task rules in working memory (to outcompete the other task). Although no external information guides task selection (i.e., no task cues are provided), task selection is constrained by instructions to select two tasks equally often and in a random (or at least unpredictable) fashion. To comply with these constraints, it has been argued that participants need to keep track of contextual information (e.g., which tasks have been performed on the last few trials) in order to generate an unpredictable and balanced task sequence across trials (Frick et al. 2019).

As switching tasks is more demanding than repeating a task (e.g., Demanet et al. 2010), adults often show a task repetition bias in VTS (e.g., Mittelstädt et al. 2018). Surprisingly, this bias is not more pronounced in children than in adults. However, children disproportionately struggle to select both tasks equally often and unpredictably, often falling back on predictable task sequences, perhaps to reduce self‐directedness demands (Frick et al. 2019, Frick et al. 2022). Critically, with enough preparation time before target onset (i.e., long inter‐trial interval), individuals can select the task they want to perform on a given trial either proactively (before target onset) or reactively (once the target has been presented). Encouraging proactive control by increasing preparation time reduces the task repetition bias in adults (e.g., Butler et al. 2011; Liefooghe et al. 2009) and 9‐ and 10‐year‐olds, but not in 5‐ and 6‐year‐olds (Frick et al. 2019), suggesting that younger children may not engage in self‐directed control as proactively as older children and adults in VTS. However, younger children select tasks in a more unpredictable fashion when they are given more time to prepare (Frick et al. 2019); thus, they may already engage control proactively but do so less efficiently than older children and adults (which may not impact the task repetition bias). Such a pattern would potentially mirror findings in contexts where cognitive control is externally driven. However, it is unclear whether and how exactly proactive control engagement may change with age in VTS.

Given that no overt response is needed before target onset, electroencephalography (EEG), which measures electrical brain activity at the scalp level, may help clarify to what extent children approach VTS proactively and how proactive preparation may differ from adults. There is clear EEG evidence for proactive motor preparation in adults in VTS. Specifically, adults show greater mu suppression on switch than repeat trials before target onset (Poljac and Yeung 2014). Mu suppression (or desynchronisation), which corresponds to a reduction in alpha (8–12 Hz) power over central channels, originates in (pre)motor cortex and is thought to reflect motor action preparation (e.g., Di Rienzo et al. 2019; Formica et al. 2021; Gable et al. 2016; Thorpe et al. 2016). Similarly, the contingent negative variation and lateralised readiness potential—two event‐related potentials associated with motor preparation—are more pronounced before target onset on task switch than task repeat trials in adults (Chen and Hsieh 2015; Henare et al. 2020; Jurczyk et al. 2022; Kang et al. 2014; Liu and Yeung 2020; Vandamme et al. 2010). Although children's proactive motor preparation in VTS has never been studied, it may not be as efficient as in adults, given prior evidence that it develops late in other tasks (e.g., Chevalier et al. 2020; Killikelly and Szuẃcs 2013; Quinzi et al. 2018).

Unlike proactive motor preparation, the neural basis of task selection in VTS has not been well documented yet. As the development of prefrontal cortex and the frontoparietal network more broadly support cognitive control improvement with age (e.g., Crone et al. 2006; Hoormann et al. 1998; Zhang et al. 2023), one may suspect that prefrontal regions are involved in task selection. Indeed, adults show greater lateral frontopolar activation in VTS than in an externally cued version of the task‐switching paradigm (Orr et al. 2019; Orr and Banich 2014). Similarly, adults’ self‐directed decisions to change their responses have been found to be supported by dorsolateral prefrontal cortex, alongside the inferior frontal junction, anterior insula and anterior cingulate cortex (Zühlsdorff et al. 2023). Neural oscillations in the delta (2–3 Hz) and theta (4–7 Hz) frequency ranges provide a major medium of communication within and across cognitive control networks, including the frontoparietal network, and are commonly associated with cognitive control performance (e.g., Cavanagh and Frank 2014; Cellier et al. 2021; Cooper et al. 2019; Gulbinaite et al. 2014; Rac‐Lubashevsky and Kessler 2018). For instance, delta/theta oscillations over frontal channels, including phase synchrony (an index of functional connectivity) between frontal and posterior channels, support externally driven proactive control (e.g., Cooper et al. 2019; McKewen et al. 2020, McKewen et al. 2021; Pscherer et al. 2023). Therefore, frontal delta and theta oscillations are likely to support proactive self‐directed task selection in VTS.

The present study used neural oscillations to investigate proactive control engagement while children and adults voluntarily switch between tasks. EEG data were collected while 5–6‐year‐olds, who have just begun using proactive control, 9–10‐year‐olds, who are more proficient at proactive control, and adults performed a VTS paradigm. Our first objective was to examine whether frontal oscillations in the delta and/or theta bands may support proactive task selection in both children and adults. If so, frontal slow oscillations should be more pronounced on switch than repeat trials. Relatedly, a second goal was to examine the extent to which task selection may be influenced by context tracking. We manipulated the difficulty of context tracking through the provision of visual support for past actions (i.e., previously selected tasks). Although context tracking may be engaged either reactively or proactively regardless of visual support, if participants *proactively* select the task based on context tracking, frontal slow oscillations should show greater power with than without visual support. In contrast, if task selection is mostly dependent on a predictable task sequence (hence failing to comply with the instruction to select tasks in an unpredictable fashion across trials), neural markers should be unaffected by visual support for past actions. Importantly, our third objective was to investigate whether potential age‐related differences in proactive control engagement with age may relate to task selection, motor preparation, or both. If self‐directed control development is driven by better task selection, we should observe less pronounced frontal slow oscillations in children than adults. If it reflects improvement in proactive motor selection, mu suppression should increase from children to adults.

## 2 Methods

### 2.1 Participants

We analysed the EEG data collected alongside the behavioural data published in Frick et al. 2022, Study 1, albeit with a smaller sample size due to unusable EEG data for some participants (seven 5–6‐year‐olds, three 9–10‐year‐olds and four adults). Participants included twenty‐five 5–6‐year‐olds (*M* = 5.7 years, SD = 0.6 year, range = 5.0–6.8, 11 females and 14 males), twenty‐seven 9–10‐year‐olds (*M* = 9.7 years, SD = 0.5 year, range = 9.0–10.9, 13 females and 14 males), and 25 adults (*M* = 22.2 years, SD = 4.5 years, range = 18.2–32.8, 12 females and 13 males). Thirty‐five children had at least one parent with a university degree, and 15 had parents with high‐school or vocational training (this information was missing for two children). All adult participants were university students. Written informed consent was obtained from all adult participants and children's parents. All children gave verbal assent, and 9–10‐year‐olds additionally provided written assent. Adult participants and children's parents were compensated £10, while children received a small, age‐appropriate prize. The study was approved by the university's ethics committee.

### 2.2 Materials and Procedure

**FIGURE 1:**
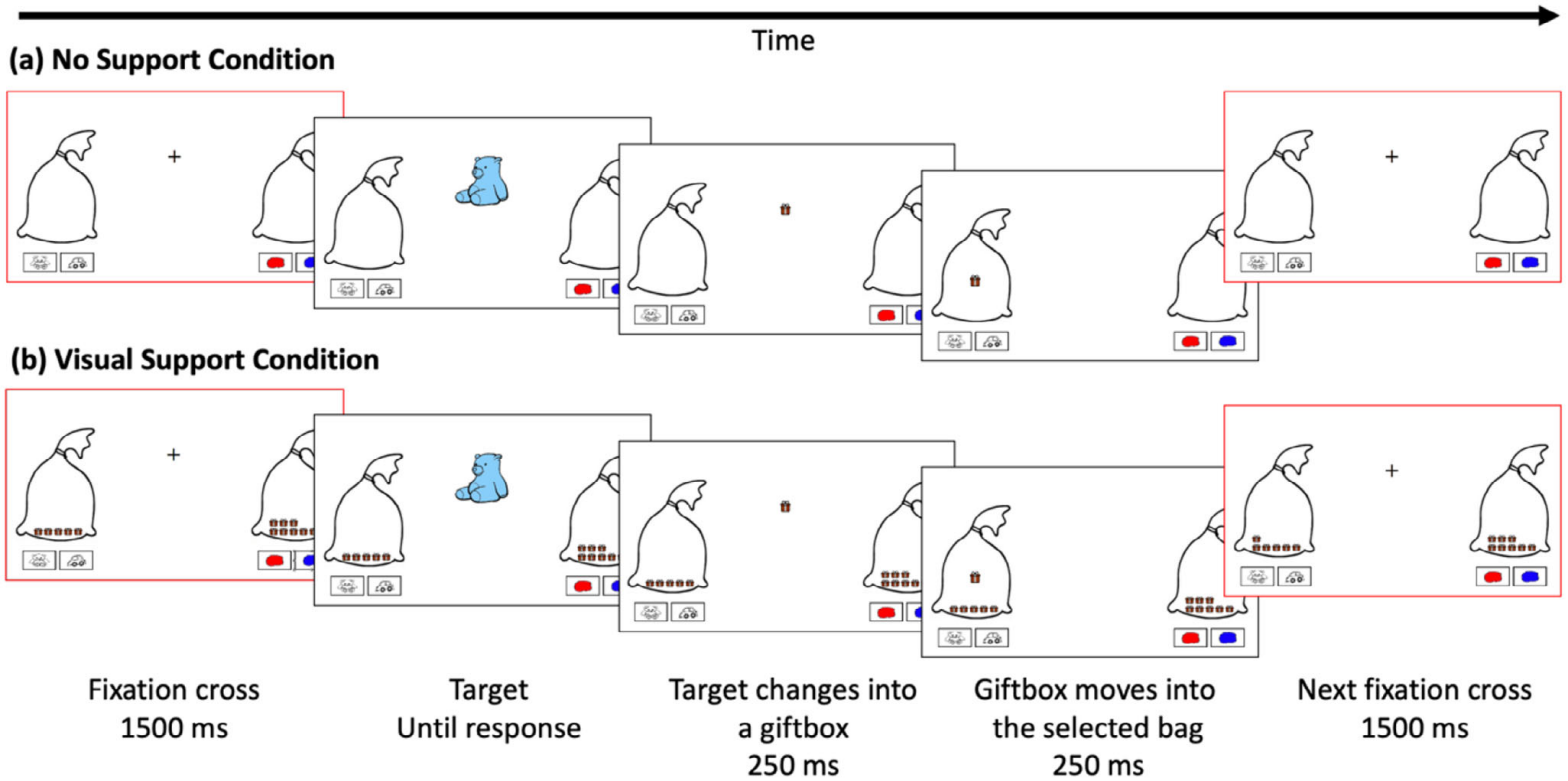
Illustration of the voluntary task switching (VTS) paradigm. Participants needed to decide whether to sort the target (e.g., blue teddy bear) by colour or shape. They responded by pressing one of the four response buttons (bottom left and right). The figure shows one trial in each of the conditions without (a) or with (b) visual support for previous actions. In this example, the target was sorted by shape. The red frames denote the time window used for EEG data analysis (i.e., fixation cross screen).

Each participant was tested individually by two experimenters (one responsible for task instructions and the other for monitoring of EEG recording) in a 90‐min session. After EEG cap application, participants completed a child‐friendly voluntary task‐switching paradigm (Frick et al. 2019) in which they had to decide on their own when to switch between two sorting tasks (colour‐ vs. shape‐sorting), but with the instructions to switch between tasks in an unpredictable fashion and to perform the two tasks roughly equally often (Figure 1). The cover story consisted of helping Santa Claus prepare for next Christmas by sorting toys into bags. On each trial, a bidimensional target (e.g., blue teddy bear) was presented centrally and participants had to match it with one of the four unidimensional response options (e.g., bear, car, red or blue). Each response option corresponded to a different button on a response box. The two buttons/response options associated with colour‐sorting were presented below the bag on one side of the screen, whereas the ones associated with shape‐sorting were presented below the bag on the other side. Dimension‐side associations were counterbalanced across participants. All four response options remained constantly visible throughout the game. Each trial started with a fixation point for 1500 ms before target onset. The target was displayed until a response was entered. Upon response entry, the toy was wrapped into a gift box (250 ms) and then sent to the selected bag (250 ms).

To convey the need to select the two tasks in an unpredictable fashion, participants were told that a thieving elf would try to steal toys for themselves. They were instructed to ‘trick’ the elf by making sure the elf could not guess which task they would play next. If children followed a predictable task sequence (e.g., alternating on every trial), the elf would be able to guess what they would do next and steal the toy. If one of five predictable task‐sequence strategies was detected, the thieving elf would come up for 500 ms after the response. The strategies involved either (a) switching only or (b) repeating the same task only over seven consecutive trials, (c) systematic alternation between task repetition and task switch over 9 consecutive trials, (d) repeated patterns of two repetitions and one switch on 11 consecutive trials, or (e) repeated patterns of three repetitions and one switch on 13 consecutive trials (see Frick et al. 2022, for further details). This procedure was used to remind participants of the need to select the task unpredictably.

Critically, in the Visual Support condition (Figure 1b), the previously sorted toys (now in gift boxes) remained visible inside the selected bags for the remainder of the game, hence allowing participants to visualise how often they had sorted targets by each dimension (colour and shape). In contrast, the previous gift boxes were not visible inside the selected bags in the No Support condition (Figure 1a), hence increasing the difficulty of keeping track of previous trials. Condition order was counterbalanced across participants.

Each condition started with two single‐task blocks in which only one dimension was relevant on all trials, which served as familiarisation/practice with each task separately. Each single‐task block involved four practice trials and 16 test trials. Then participants completed mixed‐task blocks, in which they had to switch voluntarily between the two tasks. After 16 practice trials, participants completed two blocks of 40 test trials per condition, for a grand total of 160 mixed‐task test trials.

### 2.3 Data Recording and Processing

*Behavioural data*. Response time (RT) outliers under 200 ms, above 10,000 ms or (*M* + 3SD) were removed. The remaining values were log‐transformed to correct for skew. In addition to response time and accuracy, the following three indices were computed (see Frick et al. 2022, for further details). (1) The probability of switching, *p*(switch), corresponds to the ratio of task switch trials over the total number of task switch and task repetition trials. It varies from 0 (only task repetitions) to 1 (only task switches). (2) Task sequence predictability was indexed by the number of predictable task sequences detected. Greater values correspond to more predictable task sequences. (3) Task imbalance was calculated as the difference between proportion of trials where the colour and shape tasks were performed. A score of 0 reflects perfect balance between the two tasks, whereas higher values indicate greater imbalance.

*EEG data*. A BioSemi ActiveTwo system with 64 channels (BioSemi BV, Amsterdam, Netherlands) and a 512‐Hz sampling rate was used to record EEG data. During recording, impedances were kept under 50 kΩ. The data was then processed offline with EEGLAB (Delorme and Makeig 2004), ERPLAB (Lopez‐Calderon and Luck 2014), and custom scripts. The data were re‐referenced to the average of the two mastoids, high‐pass filtered (0.1 Hz), and segmented from −2 to 2 s around target onset, excluding incorrect trials. Bad channels were automatically rejected (Kurtosis threshold = 5), and remaining bad channels were manually removed upon visual inspection. Eye‐blinks and other eye‐movement artefacts were corrected through an independent component analysis (ICA) using ADJUST (Mognon et al. 2011). Missing channels were then replaced through spline interpolation. The data were converted back to continuous data and segmented again in ERPLAB for further processing. As we focused on proactive control, segments only included the 2 s before target onset, with the initial 500 ms (i.e., the period before fixation onset) as baseline. Remaining artefacts were rejected using a 200‐ms peak‐to‐peak moving window with a 200‐Hz maximum amplitude threshold and a 100‐ms window step. All participants had at least 8 good segments per experimental cell (*M* = 26.6, SD = 6.7 at age 5–6, *M* = 32.3, SD = 5.3 at age 9–10, *M* = 34.9, SD = 4.9 in adults).

Time‐frequency decomposition was performed by convolving single‐trial data with complex Morlet wavelets: 30 frequencies increasing from 2 to 30 Hz in logarithmically spaced steps (Cohen 2014). Wavelet cycles varied from 3 to 10 as frequency increased. The data were reflected on both sides before time‐frequency decomposition to avoid edge artefacts, and reflected data were trimmed afterwards. Total power (which captures the strength of both phase and non‐phase locked activity) was normalised and decibel (dB) transformed at each frequency using the average power across all experimental cells between −1900 and −1600 ms before target onset (i.e., −400 and −100 before fixation cross onset). There is no ideal baseline in VTS, as preparation for the next trial can happen at any random time point during a trial, from the moment the previous response is entered (or even earlier) until after the next target onset (Jurczyk et al. 2022). We chose this baseline, during which the target is replaced by a giftbox and moved to the selected bag, to minimise contamination from previous trial activity while capturing all brain activity after the fixation cross‐onset. Further, it ensured comparability with previous EEG studies of VTS, which usually include the pre‐fixation period as baseline (Chen and Hsieh 2015; Henare et al. 2020; Kang et al. 2014; Vandamme et al. 2010). Although condition and trial type differences already emerged in adults, but not in children, during the baseline period (see Supporting Information), the use of a common baseline (i.e., activity averaged across all experimental cells) preserved condition and trial type differences during the fixation cross period.

Early after the fixation cross period, the data were dominated by activity in the delta and theta bands over the lateral frontal channels that peaked at channels F7 and F8 (Figure 3a). Given the lack of prior studies on this specific activity, we ran permutation tests after collapsing age groups and conditions (see Supporting Information). The permutation tests showed differences between repeat and switch trials in these frequency bands, mostly in the first half of the post‐feedback period, which confirmed the relevance of this activity. Thus, following the procedure used in prior studies (Chevalier et al. 2021; Gyurkovics and Levita 2021), power was first averaged across the 2–7 Hz frequency range and channels F7 and F8 for each time point separately. Power was then extracted for a 50‐ms window around the latency for the peak power value between −1500 (fixation cross onset) and −700 ms before target onset. This procedure allowed us to extract peak power and latency without a priori decision about the specific window to use for each age group. For mu power, selection of channels and frequency band was guided by prior studies (Formica et al. 2021; Gundlach et al. 2017; Schneider et al. 2020; Zickerick et al. 2021). The same value extraction procedure was employed, but using the 8–12 Hz range at channels C3, CP3, C4 and CP4 during the last −1000 ms before target onset. As mu suppression is generally greater over the hemisphere contralateral to the prepared action than the ipsilateral hemisphere (Meredith Weiss and Marshall 2023; Schneider et al. 2020), these four channels were categorised as either contralateral or ipsilateral channels as a function of whether the participant selected the task on the opposite or same side, respectively (e.g., if the participant selected the right‐hand task, C3 and CP3 were categorised as contralateral, and C4 and CP4 as ipsilateral channels).

### 2.4 Data Analysis

The data were analysed with a series of repeated‐measures ANOVAs including age group (younger children, older children, adults), trial type (task switch, task repetition), and condition (visual support, no support) as predictors. Models for *p*(switch), task imbalance and task sequence predictability did not include trial type. The model for mu power additionally included laterality (contralateral, ipsilateral) as predictor. Models for oscillations also included the number of trials as covariate. As our hypotheses related to power, only the models on power are reported in the results section. However, the exploratory models on peak latency are reported as Supporting Information. Post‐hoc tests were run using Tukey correction. Pearson's correlations were used to investigate the relations between behavioural and oscillation indices. For the sake of simplicity and ease of interpretation, correlations were run after collapsing values across trial types and conditions.

## 3 Results

### 3.1 Behaviour

**FIGURE 2:**
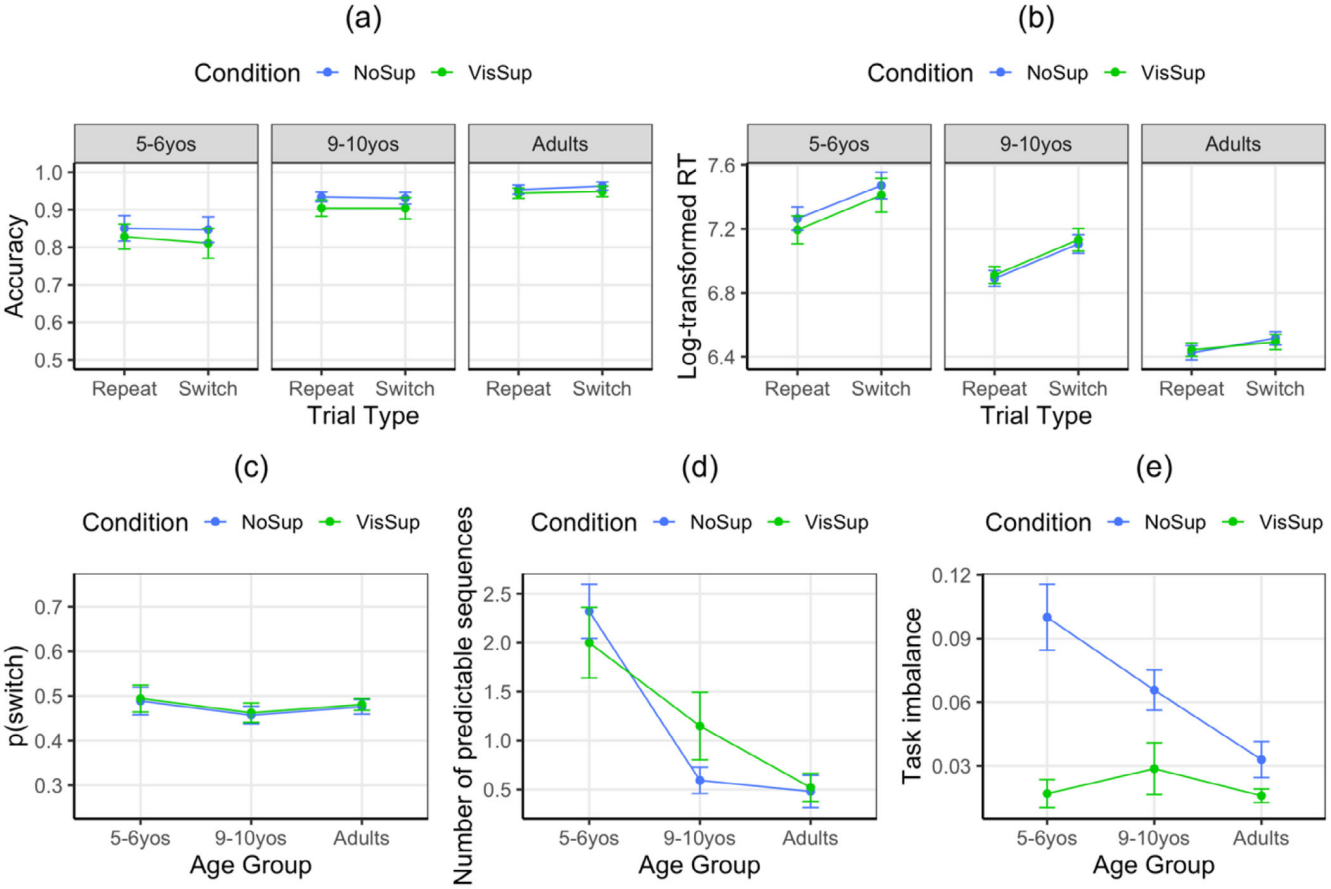
Behavioural data: (a) response accuracy, (b) response times, (c) *p*(switch), (d) number of predictable task sequences, and (e) task imbalance. NoSup, no support; VisSup, visual support. Vertical bars indicate standard errors of the means.

*Accuracy*. Accuracy (Figure 2a) varied across age groups, *F*(2, 74) = 9.65, *p* < 0.001, η_p_^2^ = 0.207, due to lower accuracy in 5–6‐year‐olds (0.83) than either 9–10‐year‐olds (0.92) or adults (0.95), *p*s < 0.008, with no difference between the two older groups, *p* = 0.426. No other effects were significant, *p*s > 0.130.

*Response times (RTs)*. The main effects of age group, *F*(2, 74) = 56.91, *p* < 0.001, η_p_^2^ = 0.606, and trial type, *F*(2, 74) = 95.71, *p* < 0.001, η_p_^2^ = 0.564, interacted with each other, *F*(2, 74) = 8.08, *p* < 0.001, η_p_^2^ = 0.179. RTs decreased with age (7.34, 7.01, and 6.47 log ms, respectively) and were slower on switch than repeat trials (7.02 vs. 6.86 log ms), *p*s < 0.001. Each age group showed a significant difference between trial types, *p*s < 0.024, although the difference was greater in children (0.20 log ms at age 5–6, 0.21 log ms at age 9–10) than adults (0.08 log ms), *p*s < 0.002, but not between the two groups of children, *p* = 0.764. No other effects were significant, *p*s > 0.497 (Figure 2b).

*P(switch)*. The probability of switching was relatively close to 0.50 in all age groups (0.49 in 5–6‐year‐olds, 0.46 in 9–10‐year‐olds, 0.48 in adults), with no significant effects of age group or condition, *p*s > 0.557 (Figure 2c).

*Task sequence predictability*. There was a main effect of age group, *F*(2, 74) = 23.08, *p* < 0.001, η_p_^2^ = 0.384. Younger children engaged in more predictable task sequences (2.16) than 9–10‐year‐olds (0.87) and adults (0.50), *p*s < 0.001. No other effects were significant, *p*s > 0.232 (Figure 2d).

*Task imbalance*. The main effects of age group, *F*(2, 74) = 5.84, *p* = 0.004, η_p_^2^ = 0.136, and condition, *F*(1, 74) = 30.27, *p* < 0.001, η_p_^2^ = 0.290, interacted with each other, *F*(2, 74) = 5.41, *p* = 0.006, η_p_^2^ = 0.128. With visual support, task imbalance was relatively low, with no differences across age groups, *p*s > 0.538. In contrast, when no support was provided, there was more task imbalance in 5–6‐year‐olds (0.10 difference in the proportions of trials where each of the two tasks was used—from 0 [the two tasks were equally frequent] to 1 [one task was used across all trials]) than adults (0.03), *p* < 0.001, with 9–10‐year‐olds (0.07) not differing significantly from the other two groups, *p*s > 0.091. Both groups of children showed less task imbalance with than without visual support, *p*s < 0.011, whereas adults did not, *p* = 0.246 (Figure 2e).

### 3.2 EEG

**FIGURE 3:**
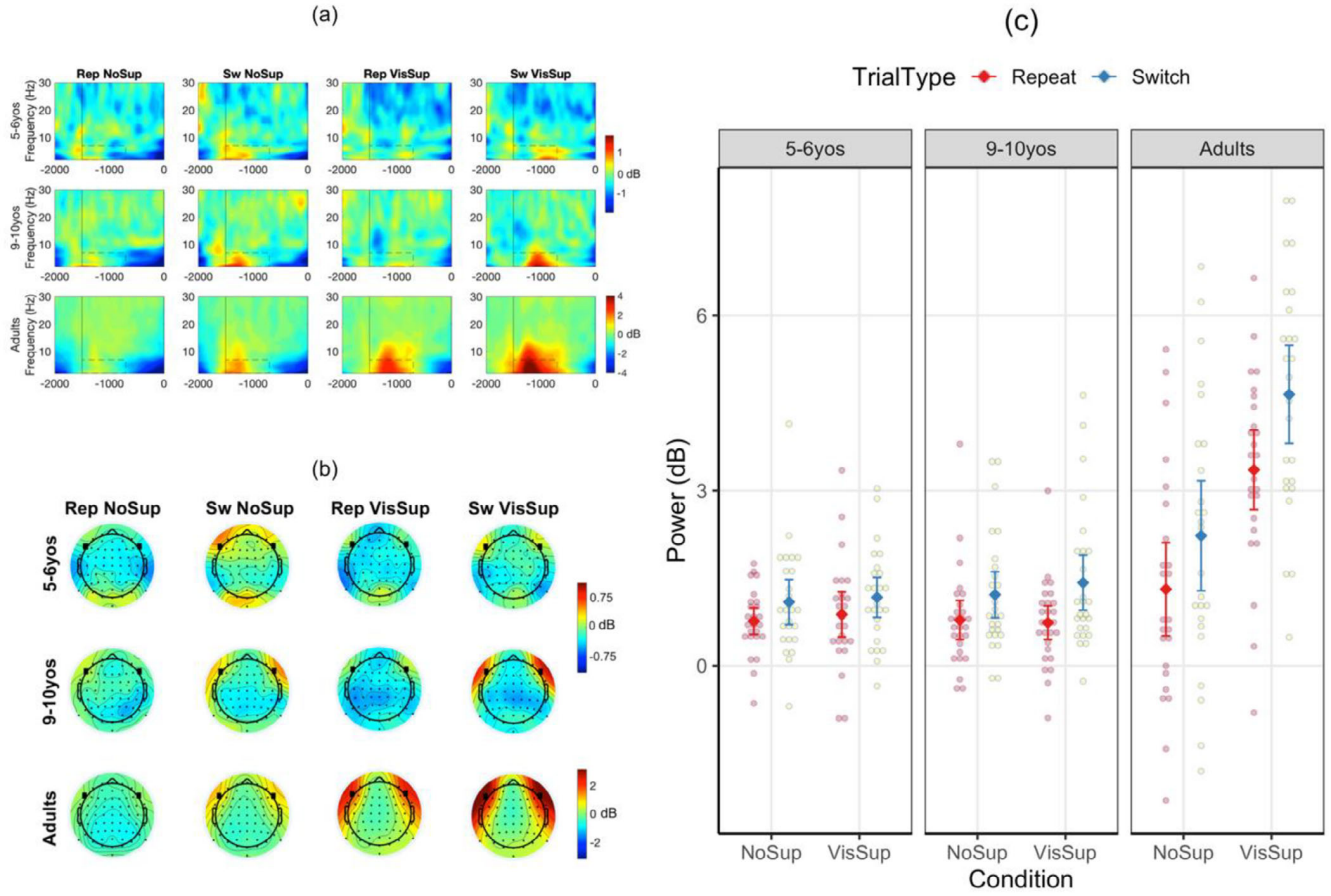
Frontolateral delta/theta power. (a) Event‐related spectral perturbations averaged across channels F7 and F8. (b) Mean topographies between −1500 and −700 ms before target onset. Channels F7 and F8 are shown in bold. The scale used in (a) and (b) differs between children and adults. (c) Mean power. NoSup, no support; Rep, repeat. Sw, switch; VisSup, visual support. Vertical errors indicate standard errors.

*Frontolateral delta/theta power*. There was a significant main effect of age group on power, *F*(2, 74) = 22.89, *p* < 0.001, η_p_^2^ = 0.382, due to higher power in adults (2.87 dB) than 5–6‐year‐olds (0.96 dB) and 9–10‐year‐olds (1.02 dB), *p*s < 0.001. Importantly, as expected if this marker relates to task selection, power was also higher on switch than repeat trials (1.95 vs. 1.29 dB), *F*(1, 74) = 84.41, *p* < 0.001, η_p_^2^ = 0.533 (Figure 3). Trial type interacted with age group, *F*(2, 74) = 10.51, *p* < 0.001, η_p_^2^ = 0.221. The difference between switch and repeat trials was significant in all age groups, *p*s < 0.017, but increased with age (0.31 in 5–6‐year‐olds, 0.56 in 9–10‐year‐olds, 1.04 in adults). Finally, power was higher with than without visual support (2.02 vs. 1.22 dB), *F*(1, 74) = 36.30, *p* < 0.001, η_p_^2^ = 0.329, which is consistent with task selection relying on context tracking. However, condition interacted with age group, *F*(2, 74) = 28.36, *p* < 0.001, η_p_^2^ = 0.434, as the effect of condition was significant in adults (2.23 dB), *p* < 0.001, but not in 5–6‐year‐olds (0.10 dB), *p* = 0.677, or 9–10‐year‐olds (0.08 dB), *p* = 0.721. No other effects were significant, *p*s > 0.139.

**FIGURE 4:**
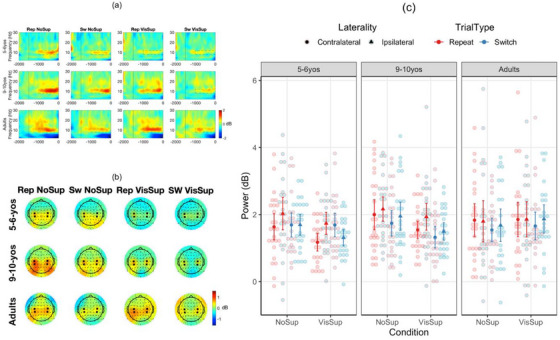
Mu suppression. (a) Event‐related spectral perturbations averaged across channels C3, C4, CP3 and CP4. (b) Mean topographies between −1000 and 0 ms before target onset. Channels C3, C4, CP3 and CP4 are shown in bold. (c) Mean power. NoSup, no support; Rep, repeat; Sw, switch; VisSup, visual support. Vertical errors indicate standard errors.

*Mu power*. As expected, mu power was lower for switch than repeat trials (1.80 vs. 1.64 dB), *F*(1, 74) = 7.48, *p* = 0.007, η_p_^2^ = 0.092, and in contra‐ than ipsilateral channels (1.64 vs. 1.80 dB), *F*(1, 74) = 13.56, *p* < 0.001, η_p_^2^ = 0.155. In addition, there was a significant age group × trial type × laterality interaction, *F*(2, 74) = 6.35, *p* = 0.002, η_p_^2^ = 0.147. Lower mu power in contra‐ than ipsilateral channels was observed in repeat trials only in 5–6‐year‐olds (−0.48) and 9–10‐year‐olds (−0.28), *p*s < 0.012, and marginally lower in switch trials in 9–10‐year‐olds (−0.19), *p* = 0.078, but there was no significant difference in adults, *p*s > 0.129 (Figure 4). Albeit unexpected, there was an effect of condition, *F*(1, 74) = 6.37, *p* = 0.013, η_p_^2^ = 0.079, that interacted with age group, *F*(2, 74) = 3.72, *p* = 0.028, η_p_^2^ = 0.091, due to lower mu power with than without visual support in both 5–6‐year‐olds (−0.29) and 9–10‐year‐olds (−0.39), *p*s < 0.037, but not adults (0.10), *p* = 0.476. Finally, there was a significant condition × trial type × laterality interaction, *F*(2, 74) = 4.36, *p* = 0.040, η_p_^2^ = 0.056. Although mu power was lower on contra‐ than ipsilateral channels in repeat trials in both conditions, the difference was greater with than without visual support (−032 vs. −0.17). No other effects were significant, *p*s > 0.060.

### 3.3 Relations Between EEG and Behaviour

Correlations between behavioural and EEG indices for each age group are shown in Table 1. Overall, behaviour showed little relation to oscillations before target onset. Lower mu power was associated with greater accuracy in 5−6‐year‐olds, *r* = ‐0.53, *p* = 0.006, which held after FDR correction.

**TABLE 1:** Correlation values between behavioural and EEG indices.

		Acc.	RT	*p*(switch)	Pred. seq.	Task imb.	δ/θ power	μ power
5‐6yos	Acc.	1	**0.48**	−0.29	−0.27	−0.25	0.04	**−0.53**
(*n* = 25)	RT	**0.48**	1	−0.23	0.11	0.32	−0.16	−0.18
	*p*(switch)	−0.29	−0.23	1	**0.42**	−0.19	0.03	0.17
	Pred. seq.	−0.27	0.11	**0.42**	1	0.09	0.14	−0.12
	Task imb.	−0.25	0.32	−0.19	0.09	1	0.02	−0.02
	δ/θ power	0.04	−0.16	0.03	0.14	0.02	1	−0.24
	*μ* power	**−0.53**	−0.18	0.17	−0.12	−0.02	−0.24	1
9‐10yos	Acc.	1	0.26	−0.04	**−0.69**	**−0.41**	−0.27	−0.05
(*n* = 27)	RT	0.26	1	−0.21	−0.22	−0.11	−0.3	−0.15
	p(switch)	−0.04	−0.21	1	−0.09	−0.19	−0.05	0.1
	Pred. seq.	**−0.69**	−0.22	−0.09	1	**0.68**	0.02	0.05
	Task imb.	**−0.41**	−0.11	−0.19	**0.68**	1	0.17	−0.08
	δ/θ power	−0.27	−0.3	−0.05	0.02	0.17	1	−0.43
	μ power	−0.05	−0.15	0.1	0.05	−0.08	**−0.43**	1
Adults	Acc.	1	−0.15	0.3	0.05	0.11	0.2	−0.21
(*n* = 25)	RT	−0.15	1	−0.15	0.18	0	−0.11	0.18
	*p*(switch)	0.3	−0.15	1	−0.15	−0.09	−0.13	−0.05
	Pred. seq.	0.05	0.18	−0.15	1	0.02	0	0.08
	Task imb.	0.11	0	−0.09	0.02	1	−0.33	0.1
	δ/θ power	0.2	−0.11	−0.13	0	−0.33	1	0.09
	μ power	−0.21	0.18	−0.05	0.08	0.1	0.09	1

*Note*: Significant *r* values (*p* < 0.050 before FDR correction) are bolded.

Abbreviations: μ, central mu; Acc., accuracy; Pred. seq., number of predictable task sequences; RT, response time; Task imb., task imbalance; δ/θ, frontolateral delta/theta.

## 4 Discussion

The present study used neural oscillations to examine proactive engagement of self‐directed control in the voluntary task‐switching paradigm in children and adults. Frontolateral delta/theta power was greater before a task switch than a task repetition in all three age groups, potentially supporting proactive self‐directed task selection in both children and adults. However, it varied as a function of visual support for past actions in adults only, suggesting that proactive task selection may rely on context tracking more in adults than children. Relative to task repetition, not only was task switching associated with greater frontolateral delta/theta power but also lower mu power before target onset in both children and adults, suggesting that even younger children proengaged control proactively in VTS. However, lateral frontal delta/theta power showed a marked increase with age, and importantly, visual support for past actions yielded different patterns of effects in children and adults, hence pointing to differences in the way proactive control was engaged across age groups. At the behavioural level, accuracy increased between 5–6‐year‐olds and the other age groups, while the switch cost on response times decreased from children to adults. Importantly, predictable task sequences decreased with age, and visual support helped children select the two tasks in a more balanced way.

Our first objective was to examine whether slow frontal oscillations may support proactive task selection. We observed that slow neural oscillations in the delta and theta frequency ranges over frontolateral channels increased from repeat to switch trials. Although this is the first time such frontolateral delta/theta oscillations are documented in VTS, this finding is consistent with the key role of frontal delta/theta oscillations in proactive control in adults (e.g., Pshecher et al., Pscherer et al. 2023). It is also in line with prior evidence for the role of lateral frontal cortex in VTS and self‐directed control more broadly in adults (Orr et al. 2019; Orr and Banich 2014; Zühlsdorff et al. 2023; but see Forstmann et al. 2006), though caution is needed when interpreting topographical information given the low spatial resolution of EEG. Frontolateral delta/theta oscillations are a likely marker of task selection processes. Not only did they vary with trial type, as would be expected given the additional demands related to deciding to switch rather than repeat a task, but they were observed before central mu oscillations, just like task selection functionally precedes motor preparation.

Our second objective was to test whether task selection may be influenced by context tracking difficulty. Provision of visual support for past actions increased frontolateral delta/theta power in adults, but not in children. However, visual support did not have any behavioural effects. This apparent discrepancy may simply reflect the fact that even without such visual support, adults efficiently tracked contextual information, as suggested by low engagement in predictable task sequences and low task imbalance. Thus, any further facilitation may have translated into greater frontolateral delta/theta power at the brain level, but not at the behavioural level. The manipulation of visual support was introduced to facilitate or encourage keeping track of prior actions (i.e., which tasks were selected on previous trials), but it may as well have reminded participants of the need to select tasks equally often and thus facilitated maintenance and use of task rules (i.e., abstract representation of the need to select both tasks equally often and unpredictably). Either way, the effect of visual support likely facilitated task selection. To our knowledge, this is the first documentation of the role of frontal delta and theta power in voluntary task switching. However, given the lack of prior studies and correlations with any of the behavioural indices, the functional interpretation of this neural marker is only speculative and will need to be confirmed in further research. Relatedly, we believe the lack of correlations between behavioural performance and frontolateral delta/theta power (and mu power) should not be interpreted as evidence suggesting that proactive control engagement does not influence self‐directed control engagement in VTS. Indeed, prior evidence showed greater performance when participants are given more time to proactively prepare (Butler et al. 2011; Frick et al. 2019; Liefooghe et al. 2009).

Our third objective was to determine whether age‐related changes in proactive engagement of self‐directed control in VTS may relate to task selection, motor preparation, or both. The results showed the overall magnitude of frontolateral delta/theta power as well as variation as a function of trial type increased with age, potentially suggesting that greater proactive task selection contributes to self‐directed control development, as assessed in VTS. This finding may reflect more efficient (e.g., greater commitment to or stronger activation of the selected task) and/or more systematic proactive task selection across trials with age, as has been previously observed in the cued task‐switching paradigm (e.g., Chevalier et al. 2020). Unlike adults, for whom trial type (and condition) differences could be detected even before the fixation cross period (see Supporting Information), trial type differences only emerged during the fixation period in children, suggesting that task selection started later in children than adults. Further, children may have relied less than adults on context tracking when selecting the upcoming task, as, unlike adults, they did not show an increase in frontolateral delta/theta power when visual support for past actions was provided, despite a greater task balance at the behavioural level. Children may have relied more than adults on predictable task sequences (e.g., switching tasks every other trial), which likely reduced the difficulty of context tracking. Indeed, visual support improved children's task balance but did not affect the number of predictable task sequences, suggesting that children similarly engaged in such predictable sequences but favoured sequence types leading to greater task balance. Consistent with the idea that visual support still influenced task selection in children, albeit in a different way than in adults, the exploratory analysis of peak latency showed that frontolateral slow power peaked later with than without visual support in children and not adults (see Supporting Information), perhaps delaying task selection due to greater consideration of task balance. Although one may argue that later peak may simply reflect the fact that more visual information was to be processed when visual support was provided, this seems unlikely given that the effect was more marked on switch than repeat trials, whereas both trial types contained the exact same amount of visual information. Visual support did not provide visual aid for the sequence of tasks previously performed, and that missing information may be important for children to select tasks based on context tracking to a greater extent than predictable task sequences. The possibility to proactively select a task without relying on context tracking (e.g., through predictable task sequences) may also explain, at least in part, why frontolateral delta/theta power did not relate to behaviour.

Frontolateral delta/theta oscillations were followed by oscillations in the mu/alpha frequency band over central and centroparietal channels in both children and adults. As expected, mu power was lower on switch than repeat trials and contralateral than ipsilateral channels before target onset, suggesting that children and adults engaged in proactive motor preparation in VTS (i.e., getting ready to respond on one of the two sides). This finding was expected in adults and is consistent with evidence for both proactive motor preparation in adults and the underlying role of neural oscillations in the mu/alpha frequency band and related to motor cortex (e.g., Pscherer et al. 2023). It is more surprising in children, given that, unlike adults, children were found not to engage in proactive motor preparation (as evidenced through the contingent negative variation—CNV) in the cued (externally driven) version of the task‐switching paradigm (Chevalier et al. 2020) and prior evidence of later development of proactive motor preparation more broadly (e.g., Killikelly and Szuẃcs 2013; Quinzi et al. 2018). However, the apparent discrepancy may stem from the fact that prior evidence was obtained through event‐related potentials (ERPs), such as the contingent negative variation or the Bereitschaftspotential component. Consistently, another study reported immature CNV but mature mu suppression patterns in 6‐ to 11‐year‐olds, suggesting that these indices may reflect different aspects of motor preparation (Bender et al. 2005). Interestingly, visual support for past actions yielded lower mu power in children, perhaps because visual support helped children commit to and further engage in motor preparation, whereas adults may either have prepared more consistently regardless of visual support or they may not have needed as much motor preparation on this task. Indeed, lower mu power was significantly associated with greater response accuracy in younger children only, although this correlation value was significantly different from older children, *z* = −1.83, *p* = 0.034, but not adults, *z* = −1.25, *p* = 0.106. Thus, although even younger children engaged in proactive motor preparation, the nature of this preparation likely refines with age.

In addition, children showed both a greater difference between contralateral and ipsilateral mu power than adults. They also showed an earlier peak latency of frontolateral delta/theta power on repeat trials than switch trials, whereas adults showed the opposite pattern. Although these findings were not expected, they may suggest a bias for task repetition in children, as has been recently reported in a visual search task (Erb et al. 2022). That is, children may prepare more fully for task repetitions, whereas adults may show greater preparation for task switches than repetitions. This may reflect more optimal adjustment of cognitive control engagement as a function of task demand variations with age (Chevalier 2015). However, these unexpected findings will need to be confirmed in the future.

Together, the present findings provide direct, neural evidence that children engaged control proactively in VTS, which is consistent with prior behavioural findings in older children and adults and disambiguates mixed behavioural findings in younger children (Frick et al. 2019). These findings go beyond previous behavioural information by shedding light on how children engage these proactive control processes and how they differ from adults. They are remarkable given young children struggle with proactive control when control is externally driven, which is often assumed to reflect children's relatively limited working memory to face the additional demands of proactiveness (for evidence of relations between proactive control and working memory in childhood, see Gonthier et al. 2019; Kubota et al. 2020; Troller‐Renfree et al. 2020; Zhou et al. 2023). The extra cognitive demands related to self‐directedness did not deter children from engaging control proactively, which does not appear to depend exclusively on the amount of cognitive demands. Self‐directedness likely provides a greater incentive for proactiveness than external cues, as keeping track of past actions and anticipating future ones likely prompts proactive control engagement (despite the additional demands), and conversely, some form of proactiveness may be needed in order to keep track of past actions and anticipate future ones. The present findings raise the possibility that increasing self‐directedness and proactiveness with age—two of the main developmental trends in cognitive control development—may be intrinsically tied, although further research is needed to explore the exact nature of these potential ties both in VTS and beyond. For instance, they may stem in part from both control forms relying on the same factors, including working memory, prospective memory, and abstract goal representations (Frick et al. 2019; Mahy et al. 2015; Munakata et al. 2012).

In conclusion, the present findings pointed out the potential role of frontolateral delta/theta power in proactive task selection in the voluntary task‐switching paradigm. They showed that 5‐ and 6‐year‐olds engage in self‐directed control in a proactive way in this paradigm, although they do so differently than adults. As such, they represent an important first step towards better understanding the neurocognitive processes underlying self‐directed control development. Further research is needed to examine how exactly self‐directed and proactive control developments may build on each other.

## Conflicts of Interest

The authors declare no conflicts of interest.

## Supporting information




**Supporting File 1**: desc70073‐sup‐0001‐SuppMat.docx

## Data Availability

The data and preprocessing scripts are available on the Open Science Framework https://osf.io/7rqdk/?view_only=f256ee64e25240b882b1dde3450e5aeb.
