# Supplementary material for: Neural Oscillatory Markers of Voluntary Task Switching: Proactive Engagement of Self‐Directed Control in Children and Adults
Source: Dev Sci. 2025 Sep 8;28(6):e70073. doi: 10.1111/desc.70073 (PMC12417625; doi:10.1111/desc.70073)
Supplement: Supplementary file 1 — Supporting File 1: desc70073‐sup‐0001‐SuppMat.docx [file DESC-28-e70073-s001.docx]

**Supplementary material**

Section A. Baseline period EEG analysis

Power in the delta/theta (2-7 Hz) and alpha (8-12 Hz) was averaged over the whole baseline period (i.e., -400 m to -100 ms before fixation cross onset) and across channels F7 and F8, and C3, C4, CP3 and CP4, respectively. Two ANOVAs were run with age group, condition, and trial type as predictors, and number of good segments as covariate.

The ANOVA on delta/theta power showed main effects of age group, *F*(2, 74) = 16.43, *p* < .001, η_p_^2^ = .307, condition, *F*(1, 74) = 18.5, *p* < .001, η_p_^2^ = .200, and trial type, *F*(1, 74) = 10.45, *p* = .001, η_p_^2^ = .123, as well as an age group × condition interaction, *F*(2, 74) = 11.42, *p* < .001, η_p_^2^ = .236, and an age group × trial type interaction, *F*(2, 74) = 13.32, *p* < .001, η_p_^2^ = .265. Delta/theta power was lower without than with visual support in adults (-1.15 vs 0.43 dB), *p* < .001, but not in 5-6-year-olds (-0.23 vs -0.02 dB) or 9-10-year-olds (-0.12 vs -0.07 dB), *p*s > .396. Similarly, it was lower on task repeat than task switch trials in adults (-0.78 vs 0.06 dB), *p* < .001, but not in 5-6-year-olds (-0.07 vs -0.18 dB) or 9-10-year-olds (-0.12 vs -0.08 dB), *p*s > .448. No other effects were significant, *p*s >.539.

The ANOVA on alpha power showed a main effect of age group, *F*(2, 74) = 3.29, *p* = .043, η_p_^2^ = .082, and an age group × condition interaction, *F*(2, 74) = 7.45, *p* = .001, η_p_^2^ = .168. Alpha power was lower without than with visual support in adults (-0.31 vs 0.12) *p* < .001, but not in 5-6-year-olds (-0.11 vs -0.10 dB) or 9-10-year-olds (0.02 vs -0.18 dB), *p*s > .083. No other effects were significant, *p*s > .068.


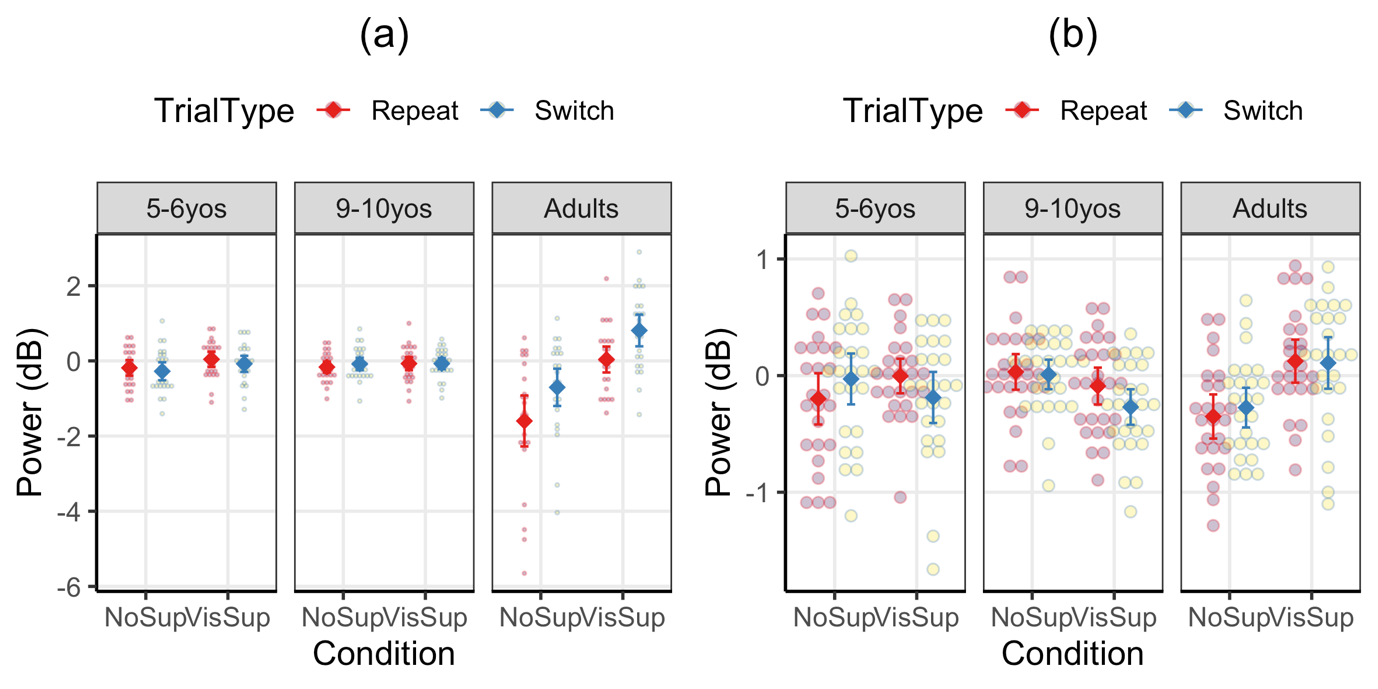


*Figure S1*. (a) Mean delta/theta power at channels F7 and F8 , and (b) alpha power at channels C3, C4, CP3 and CP4 during the baseline (-400 to -100 ms before fixation cross onset. Rep = Repeat. Sw = Switch. NoSup= No support. VisSup = Visual support. Vertical errors indicate standard errors. Effects of condition (both frequency bands) and trial type (delta/theta power only) were already observed in adults, but not in children.

Section B. Cluster-based permutation tests

To identify significant differences in EEG activity between switch and repeat trials, we ran permutation tests on participants from all age groups. We averaged data across channels F7 and F8 and conditions, and conducted the analysis on 2-30 Hz frequencies using the 1500 ms window before stimulus onset. We ran 1000 permutations to estimate the distribution of test statistic under the null hypothesis with a threshold of *p* < .05. Cluster-based correction was performed to correct for multiple comparisons. Figure S1 shows the difference in EEG activity between switch and repeat trials for each age group separately (a) and for all participants together (b). Significant differences are marked by black contours on the panel for all participants. Significant differences between switch and repeat trials were observed for a large cluster mostly spanning the delta and theta bands during the first half of the fixation cross period.


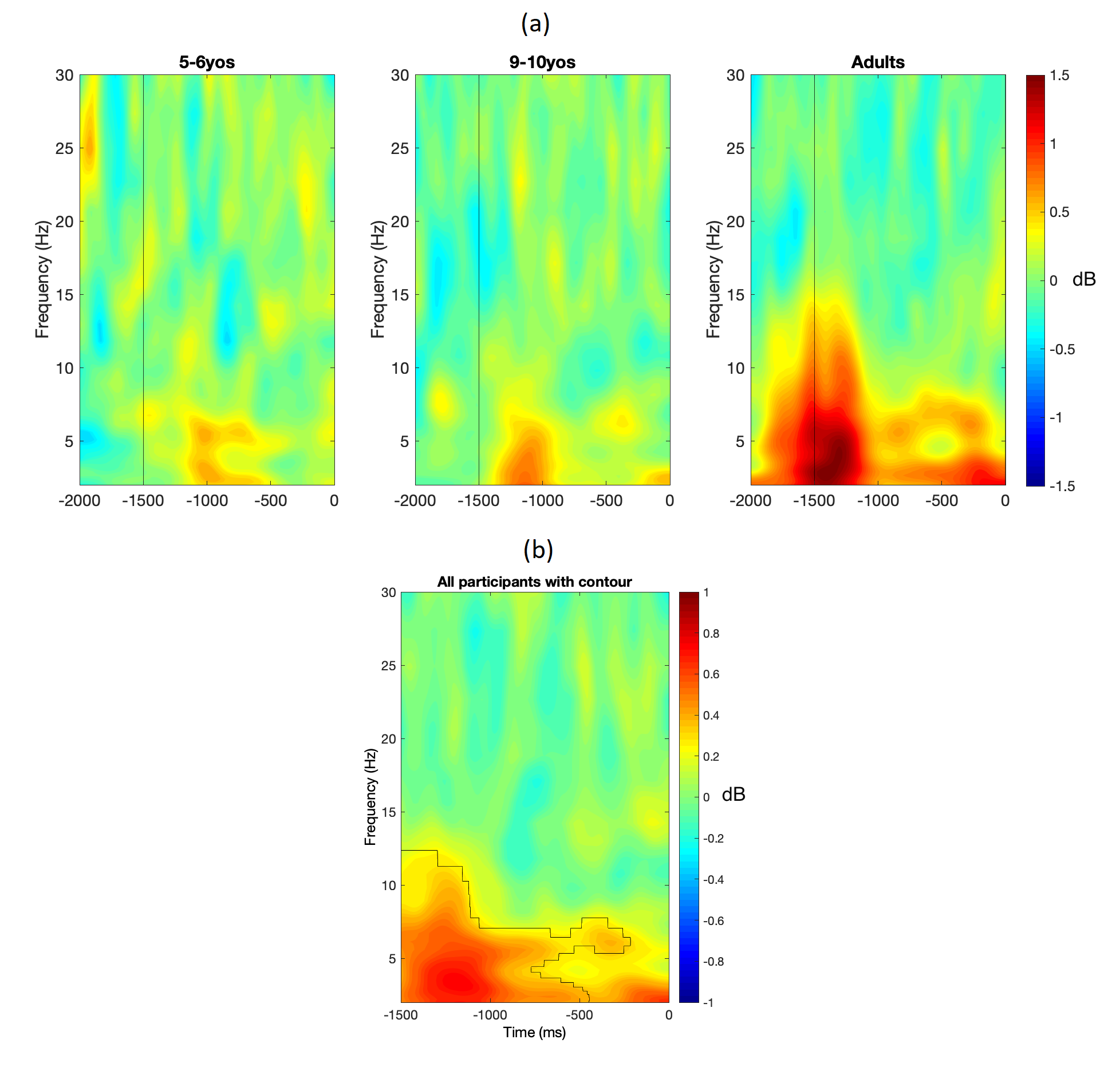


*Figure S2.* Switch minus repeat trials at channels F7-F8 (averaged together) after collapsing the visual support and no support conditions. (a) Difference in EEG activity in each age group separately. (b) Difference in EEG activity for all participants together. The black contour in (b) delineates statistically significant differences, as evidenced by permutation tests. Note that permutation tests were run on all participants together (not by age group).

Section C. Analysis of peak latency during the fixation cross period

*Frontolateral delta/theta power peak latency.* The model showed a main effect of condition, *F*(1, 74) = 32.94, *p* < .001, η_p_^2^=.120, that interacted with age group, *F*(2, 74) = 4.06, *p* = .020, η_p_^2^ = .030 (Figure S3a). Frontolateral delta/theta power peaked later with than without visual support in both 5-6-year-olds (-1067 vs -1260 ms), *p* < .001, and 9-10-year-olds (-1073 vs -1236 ms), *p* < .001, but not adults (-1187 vs -1227 ms), *p* = .322. Further, trial type interacted with both age group, *F*(2, 74) = 9.56, *p* < .001, η_p_^2^ = .040, and condition, *F*(2, 74) = 4.33, *p* < .040, η_p_^2^ = .010. Latency peaked earlier on switch than repeat trials in adults (-1245 vs -1169 ms), *p* = .007, while the reverse was observed in 5-6-year-olds (-1116 vs -1211 ms), *p* = .001, and the difference was not significant in 9-10-year-olds (-1142 vs -1167 ms), *p* = .354. Although it peaked later with than without visual support for both trial types, *p*s < .002, the difference was greater on switch than repeat trials (171 vs 94 ms). No other effects were significant, *p*s > .190.

*Mu power peak latency*. The model showed a main effect of age group, *F*(2, 74) = 6.47, *p* = .002, η_p_^2^ = .149, that interacted with laterality, *F*(1, 74) = 4. 51, *p* = .019, η_p_^2^ = .10 (Figure S3b). Mu power peaked earlier for adults than 9-10-year-olds (-576 vs -457 ms), *p* = .001. It peaked earlier for contra- than ipsilateral channels in 9-10-year-olds (-483 vs -431 ms), *p* = .017, with no difference in the other age groups, *p*s > .125. Although there was also a trial type × laterality interaction, *F*(1, 74) = 4.96, *p* = .029, η_p_^2^ = .063, none of the pairwise comparisons were significant, *p*s > .114. No other effects were significant, *p*s > .077.


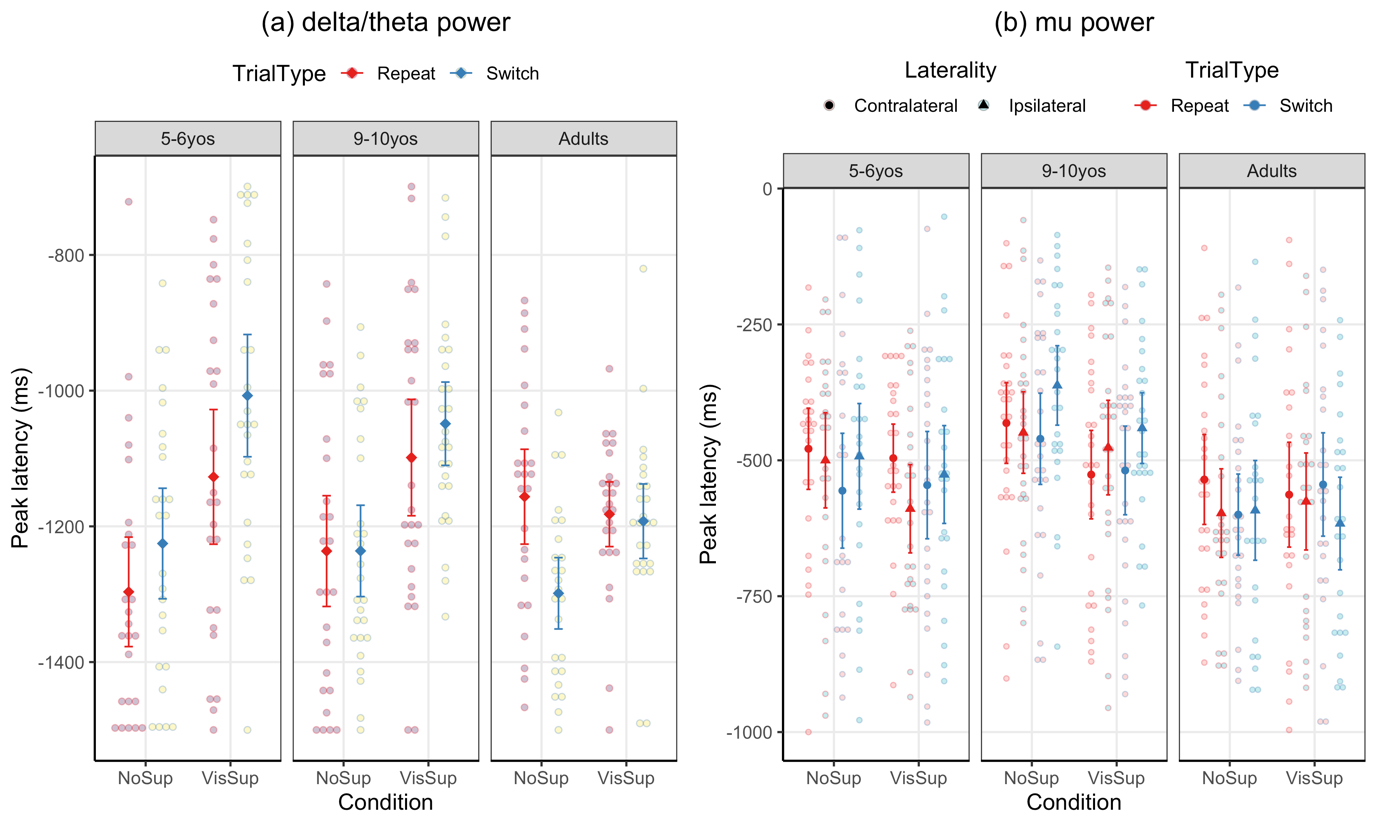


*Figure S3*. Mean peak latency of (a) frontolateral delta/theta power and (b) mu power. Rep = Repeat. Sw = Switch. NoSup= No support. VisSup = Visual support. Vertical errors indicate standard errors.
